# Relevance of ERCC1, RRM1 and BRCA1 in surgically treated non-small cell lung cancer patients

**DOI:** 10.1186/1878-5085-5-S1-A27

**Published:** 2014-02-11

**Authors:** Martin Pesta, Ondrej Topolcan, Vlastimil Kulda, Ondrej Fiala, Gabriela Krakorova, Radim Cerny, Milos Pesek

**Affiliations:** 1 Biology department, Faculty of Medicine and University hospital in Pilsen, Charles University in Prague, Czech Republic; 2 Internal Medicine II, Faculty of Medicine and University hospital in Pilsen, Charles University in Prague, Czech Republic; 3 Biochemistry department, Faculty of Medicine and University hospital in Pilsen, Charles University in Prague, Czech Republic; 4 Oncology department, Faculty of Medicine and University hospital in Pilsen, Charles University in Prague, Czech Republic; 5 Tuberculosis and Respiratory Diseases, Faculty of Medicine and University hospital in Pilsen, Charles University in Prague, Czech Republic

## Scientific objectives

Chemotherapy has been an important modality of treatment of NSCLC. Recent studies have shown that assessment of predictive molecular markers could be helpful for estimation of response rate to chemotherapy. The aim of our study was to assess relation of mRNA levels of DNA repair genes ERCC1, RRM1 and BRCA1 in surgically resected tumor tissue in patients who received adjuvant chemotherapy to disease free interval (DFI) and overall survival (OS). We investigated if potential residual tumor cells after resection reflect properties of a primary tumor and response to chemotherapy according to level of predictive markers with the respect to current knowledge.

## Technological approaches

We studied group of 90 patients with NSCLC, who had undergone curative lung resection, 59 of them were subsequently treated with adjuvant chemotherapy, DFI and OS were evaluated only in this subgroup. Quantitative estimation of mRNA of selected genes in paired (tumor and control) lung tissue samples was performed by RT real-time PCR.

## Results interpretation

We found lower mRNA expression of ERCC1 (p<0.001) and RRM1 (p=0.023) in NSCLC tumor tissue compared to normal lung tissue. Comparing expression in histological subtypes we recorded higher mRNA expression of ERCC1 (p=0.021), RRM1 (p=0.011) and BRCA1 (p=0.011) in adenocarcinoma than in squamous cell carcinoma (SCC). The differences in DFI and OS were found only in specific subgroups according to tumor type and stage. We found longer OS in patients with adenocarcinoma with higher expression of RRM1 mRNA (p=0.002). We found longer OS in patients with SCC with higher expression of BRCA1 mRNA (p=0.041). In NSCLC patients of stage III, we found longer DFI in patients with higher expression of RRM1 (p=0.004) and ERCC1 (p=0.038).

## Outlook and expert recommendations

Patients who had been treated with adjuvant chemotherapy and had shown lower expression of repair genes had adverse prognosis. We observed that the assessment of DNA repair gene level in primary tumor treated by surgical resection had prognostic significance and did not predict response to adjuvant chemotherapy.

